# Circumstances and toxicology of violence-related deaths among young people who have had contact with the youth justice system: a data linkage study

**DOI:** 10.1186/s12889-021-12244-z

**Published:** 2021-12-03

**Authors:** Melissa Willoughby, Jesse T. Young, Katie Hail-Jares, Matthew J. Spittal, Rohan Borschmann, George Patton, Susan M. Sawyer, Emilia Janca, Linda Teplin, Ed Heffernan, Stuart A. Kinner

**Affiliations:** 1 Melbourne School of Population and Global Health, The University of Melbourne, Justice Health Unit, Level 4, 207 Bouverie Street, Carlton, Parkville, Victoria 3053 Australia; 2 Centre for Adolescent Health, Murdoch Children’s Research Institute, Parkville, Victoria Australia; 3 School of Population and Global Health, The University of Western Australia, Perth, Western Australia Australia; 4 National Drug Research Institute, Curtin University, Perth, Western Australia Australia; 5 School of Criminology and Criminal Justice, Griffith University, Brisbane, Queensland Australia; 6 Health Service and Population Research Department, Institute of Psychiatry, Psychology & Neuroscience, King’s College London, London, UK; 7 Melbourne School of Psychological Sciences, The University of Melbourne, Parkville, Victoria Australia; 8 Department of Paediatrics, The University of Melbourne, Parkville, Victoria Australia; 9 The Nossal Institute for Global Health, The University of Melbourne, Parkville, Victoria Australia; 10 Feinberg School of Medicine, Northwestern University, Chicago, USA; 11 Forensic Mental Health Group, Queensland Centre for Mental Health Research, Brisbane, Australia; 12 Faculty of Medicine, The University of Queensland, Brisbane, Australia; 13 Queensland Forensic Mental Health Service, Queensland Health, Brisbane, Australia; 14 Mater Research Institute-UQ, University of Queensland, Brisbane, Queensland Australia; 15 Griffith Criminology Institute, Griffith University, Brisbane, Queensland Australia; 16 School of Public Health and Preventive Medicine, Monash University, Melbourne, Victoria Australia

**Keywords:** Homicide, Violence, Death, Toxicology, Young people, Medio-legal

## Abstract

**Background:**

Young people who have had contact with the youth justice system have an increased risk of dying from violence. Examining the context of violence-related deaths is essential in informing prevention strategies. We examined the circumstances and toxicology of violence-related deaths among young people who have had contact with the youth justice system in Queensland, Australia.

**Methods:**

This data linkage study linked youth justice records from Queensland, Australia (30 June 1993-1 July 2014) on 48,670 young people to national death and coroner records (1 July 2000-1 January 2017). Circumstances and toxicology of deaths were coded from coroner’s records. We calculated the incidence of violence-related deaths that were reported to a coroner. Fisher’s exact tests were used to examine crude differences in the circumstances and toxicology of violence-related death, according to sex and Indigenous status.

**Results:**

There were 982 deaths reported to a coroner in the cohort. Of which, 36 (4%) were from violence-related causes (incidence: 6 per 100,000 person-years, 95% confidence interval: 4-8). People who died from violence were most frequently male (*n* = 28/36; 78%), and almost half were Indigenous (*n* = 16/36; 44%). The majority of violence-related deaths involved a weapon (*n* = 24/36; 67%), most commonly a knife (*n* = 17/36; 47%). Compared to men where the violent incident was most frequently preceded by an altercation (*n* = 12/28; 43%), for women it was frequently preceded by a relationship breakdown or argument (*n* < 5; *p* = 0.004). Substances most commonly present in toxicology reports were cannabis (*n* = 16/23; 70%) and alcohol (*n* = 15/23; 65%).

**Conclusions:**

Therapeutic alcohol and other drug programs, both in the community and detention, are likely important for reducing violence-related deaths among young people who have had contact with the youth justice system. The majority of violence-related deaths among women were in the context of intimate partner violence, indicating the urgent need for interventions that prevent intimate partner violence in this population. Diversion programs and increased investment in health and social services may reduce the overrepresentation of Indigenous people in the youth justice system and in violence-related deaths.

**Supplementary Information:**

The online version contains supplementary material available at 10.1186/s12889-021-12244-z.

## Background

Young people who have had contact with the youth justice system (hereafter referred to as justice-involved young people) are more likely to die from violence compared to the general population of the same age [1]. Although limited, research on violence-related deaths among justice-involved young people has largely drawn upon United States (US) based samples [1, 2], the extent that these risks are shared in other countries is unknown. Studies from Australia [3] and Finland [4] report proportions of deaths due to violence (3-12%) that are considerably lower than in the US (48-68%) [1, 2]. Examining why, where and how these deaths occur is important to understand these international differences and to inform targeted violence prevention strategies.

To date, only one study, which included adults released from prison in the US, has examined the circumstances of violence-related death among people involved in the criminal justice system [5]. This study found that common circumstances included being involved in a crime, or an argument with a person known to the deceased, before death [5]. To our knowledge, no previous studies have examined the circumstances of violence-related deaths among justice-involved young people. This is surprising given that young people are more likely to be involved in the criminal justice system [6], and are more likely to be victims of violence [7], than other age groups.

Evidence from the general population may provide some useful insights into the likely circumstances of violence-related deaths among justice-involved young people. In the general population, substance use, particularly alcohol use, is associated with being both a victim and perpetrator of violence [8]. The majority of victims and perpetrators of violence are young men [7], while women constitute the majority of victims of family, intimate partner and sexual violence [7]. People who are socioeconomically deprived, marginalised (e.g., Indigenous people, Black people) [9], or criminalised (e.g. people who use drugs) [8] are disproportionately impacted by violence victimisation. These groups are also overrepresented in the youth and adult criminal justice systems [10]. Given this, preventing violence among justice-involved young people is likely an important target for addressing health inequity.

The circumstances and toxicology (the measurement and analysis of substances present in a person’s body [11]) of violence-related deaths can be explored using data from coroner’s investigations. Coroner’s data have been used to examine the circumstances and toxicology of violence-related deaths in the general population in Australia [12] and internationally [8, 13]. Building upon these methods, we aimed to 1) describe the circumstances and toxicology of violence-related deaths reported to an Australian coroner among justice-involved young people, and 2) examine whether the circumstances and toxicology of these deaths differed by sex and/or Indigenous status.

## Methods

### Study design

We conducted a retrospective data linkage study of young people who had contact with the youth justice system in Queensland, Australia from 30 June 1993 to 1 July 2014. We examined deaths occurring after contact with the youth justice system that were reported to an Australian coroner from 1 July 2000 to 31 January 2017.

### Data sources and linkage process

Data on all young people (aged 10-18 years) who had contact with the youth justice system in Queensland from 30 June 1993 to 1 July 2014 were retrieved from youth justice records. Youth justice system contact included being charged with an offence, with or without being found guilty, or being found guilty of an offence and subsequently being sentenced to a community-based or a custodial order (i.e., youth detention). During the study period, Youth Justice Queensland was responsible for young people aged 10 to 16 years who came in contact with the youth justice system [14]. Once sentenced, some young people could remain in youth detention or under youth justice supervision in the community after the age of 16 [14]. For this reason, young people up to the age of 18 years at baseline are included in our cohort.

Youth justice records were probabilistically linked, with subsequent clerical review, to death records from the National Death Index (NDI) [15] (30 June 1993-31 January 2017) and to the National Coronial Information System (NCIS) [16] (1 July 2000-31 January 2017) using participants’ name, sex, date of birth, and all known aliases. Youth justice records were also probabilistically linked with adult correctional records (1 January 1994-31 December 2016), which provided information on subsequent incarcerations within the adult system. This linkage methodology is considered the ‘gold standard’ approach to accurately and reliably identify deaths among people involved in the criminal justice system in Australia [17].

The NCIS contains records from all coroner’s investigations in Australia since 1 July 2000 (1 January 2001 for Queensland) and in New Zealand since July 2007 [16]. In Australia, some deaths are investigated by a coroner in order to determine the identity of the decedent, the cause of death, and to inform recommendations to prevent future deaths of a similar cause and circumstance [16]. While criteria for reporting a death to the coroner vary between jurisdictions, typically, reportable deaths are unexpected; occur in violent and/or unnatural circumstances; occur as a result of, or during, a medical or surgical procedure; occur in State care or custody; or where the person’s identity or cause of death is unknown [16]. Each death in the NCIS can contain up to four full-text reports from the coroner’s investigation including: a police summary of circumstances, autopsy and toxicology reports, and the coroner’s finding [16]. The NCIS also contains pre-coded fields on demographic (e.g., age, sex, Indigenous status) and mortality (e.g., cause and location of death) characteristics [16]. Only deaths where the coroner’s investigation was complete at the time of data extraction (i.e., ‘closed records’ [16]) were included.

### Primary outcome

We examined violence-related deaths reported to an Australian coroner that occurred in the community following contact with the youth justice system. Violence-related deaths included those where the underlying cause of death was coded using the following International Classification of Diseases Tenth Edition codes in either the NCIS or the NDI: T74, X85–Y09, Y87.1, Y35, Y89.0. Additionally, we included deaths where the ‘intent’ of death in the NCIS was pre-coded as either 1) ‘assault’, or 2) ‘legal intervention’ (i.e., the killing of a person by a police officer acting in the course of duty) where the officer was in close contact with the deceased.

### Measures

Age at death (< 19/19-24/≥25 years), sex (male/female), and most serious youth justice contact over the study period (charge without an order/order without detention/detention) were obtained from youth justice records. To minimise under-ascertainment, a common issue with coding of Indigenous status [18], a person was considered Indigenous if they were coded as such in any death or correctional data source. Employment status (employed/unemployed/other/not reported) and relationship status (married or de facto relationship/not in a relationship/not reported) were extracted from pre-coded NCIS fields.

Other information on the circumstances of the violence-related death were coded by the lead author from full-text reports in the NCIS using quantitative manifest content analysis [19]. We used a combination of a priori and emergent codes. The codes for the perpetrator’s sex (0 = male/1 = female/2 = not reported) and the relationship of the perpetrator to the deceased (0 = stranger/1 = friend or acquaintance/2 = intimate partner/3 = family member/4 = not reported) were determined a priori. Open text coding was used to record the number of perpetrators, the date and location of the violent incident, and type of weapon used. Codes relating to the events preceding the fatal violent event emerged during the analytical process. If reported, the text describing the events that preceded the fatal violence was extracted and later reviewed to identify codes. These codes were grouped into the category ‘events preceding the fatal violent event’. All substances present in the toxicology reports uploaded to the NCIS were recorded.

### Statistical analysis

Frequencies and proportions were used to describe the data. As distributions were skewed, medians and interquartile ranges (IQR) are reported. Where possible, we compared crude differences in the circumstances and toxicology violence-related deaths by sex and Indigenous status using Fisher’s exact test (results not presented in the tables due to the small number of deaths in some cells). We calculated the crude rate of violence-related death per 100,000 person-years of observation in the community, excluding time spent in youth detention or adult incarceration. As the circumstances and toxicology of violence-related deaths are likely different in the incarceration environment compared to the community, fourteen deaths, none from violence-related causes, that occurred during a period of incarceration were excluded. Table ambiguation by standard cell suppression (cells *n* < 5) was employed to avoid potential re-identification. All analyses were conducted in Stata/SE Release 15 [20].

## Results

The cohort included 48,670 young people (aged 10-18 years at the start of the study period), who were followed for a total of 623,823 person-years in the community (median follow-up per person = 14 years; IQR 8-18). Over the study period, the most serious youth justice contact experienced by the cohort was frequently a charge (*n* = 28,250, 58%), followed by an order (*n* = 12,878, 26%), and detention (*n* = 7542, 16%). There were 1261 deaths, of which 982 (78%) occurred in the community and had a record on the NCIS (Supplementary Fig. S1). Thirty-six (4%) of these deaths were from violence-related causes. The rate of violence-related deaths that were reported to a coroner was 6 deaths per 100,000 person-years (95% confidence interval: 4-8). Of the cohort whose most serious youth justice contact was a charge, 19 (0.1%) died from violence. Of those whose most serious contact was an order or detention, 7 (0.105%) and 10 (0.1%) died from violence, respectively. The most common other cause of death was suicide (*n* = 363; 37%), followed by transport accidents (*n* = 190; 19%) (Supplementary Table S1).

### Characteristics of people who died from violence-related causes

The majority of the 36 people who died from violence were male (*n* = 28; 78%) and, where employment status was known, not employed at the time of death (*n* = 15; 42%) (Table 1). Just under half were Indigenous (*n* = 16; 44%), despite Indigenous people comprising only 27% of the cohort. At the time of death, a higher proportion of women who died from violence (*n* = 5; 63%) than men who died from violence (*n* = 6; 21%; *p* = 0.038) were married or in a de facto relationship. At the end of the study period, the most serious youth justice contact experienced by those who died from violence was most frequently a charge only (*n* = 19, 53%), followed by detention (*n* = 10, 28%), and an order without detention (*n* = 7, 19%). The median age at death for those who died from violence was 24 years (IQR 20-28 years; range 14-32 years). Eleven violence-related deaths (31%) had secondary causes of death that were not directly related to injuries sustained during the violent incident. These were most frequently substance-related causes (*n* = 10; 28%).

**Table 1:** Demographic characteristics of young people who had contact with the youth justice system

	Person-years of follow-up	Violence-related deaths (***n*** = 36)	Other causes of death (***n*** = 946)	Whole cohort (***N*** = 48,670)
		n (%)	n (%)	N (%)
**Age at death (years)**				
< 19	6886	9 (25)	185 (20)	1830 (4)
19-24	95,500	11 (31)	323 (34)	13,975 (29)
≥ 25	521,436	16 (44)	438 (46)	32,865 (68)
**Sex**				
Male	474,803	28 (78)	805 (85)	36,773 (76)
Female	149,021	8 (22)	141 (15)	11,897 (24)
**Indigenous status**				
Non-Indigenous	465,957	20 (56)	700 (74)	35,420 (73)
Indigenous	157,866	16 (44)	246 (26)	13,250 (27)
**Most serious youth justice contact**^**1**^				
Charge without an order	359,903	19 (53)	447 (47)	28,250 (58)
Order without detention	177,589	7 (19)	275 (29)	12,878 (26)
Detention	86,332	10 (28)	224 (24)	7542 (16)
**Relationship status at time of death**				
Married or in a de facto relationship	^2^	11 (31)	199 (21)	^2^
Not in a relationship	^2^	9 (25)	506 (53)	^2^
Not reported	^2^	16 (44)	241 (25)	^2^
**Employment status at time of death**				
Employed	^2^	5 (14)	180 (19)	^2^
Not employed	^2^	15 (42)	448 (47)	^2^
Other^3^	^2^	5 (14)	80 (8)	^2^
Not reported	^2^	11 (31)	238 (25)	^2^

1. At the end of the study period. 2. Only available for people who died during the study period. 3. Other includes: student, receiving social welfare payments, and home duties

### Circumstances of the violent incident

The majority of the 36 violence-related deaths involved the use of a weapon (*n* = 24; 67%), most commonly a knife (*n* = 17; 47%; Table 2). The violent event most commonly occurred in a private location (*n* = 20; 56%). Where the date of the violent event was recorded (*n* = 31; 86%), it often occurred on a Friday (*n* = 9; 25%), followed by a Sunday (*n* = 7; 19%). For men, fatal violence was frequently preceded by a physical or verbal altercation (*n* = 12; 43%), whereas for women, it was frequently preceded by a relationship breakdown or argument (*n* < 5; *p* = 0.004). The circumstances of death did not differ by Indigenous status (*p* > 0.05). There were no deaths from legal intervention in the cohort.

**Table 2:** Circumstances and toxicology of violence-related deaths reported to a coroner among justice-involved young people

	Frequency (n)	Proportion (%)
**CIRCUMSTANCES OF DEATH (*n*** **= 36)**		
**Events preceding the fatal violent event**		
Being involved in a physical or verbal altercation	14	39
Relationship breakdown or argument with partner	< 5	< 14
Deceased was involved in a crime	< 5	< 14
Not reported	15	42
**Use of weapon**		
Assault with a knife	17	47
Assault with another weapon	6	17
Bodily force	6	17
Firearm	< 5	< 14
Not reported	6	17
**Number of perpetrators of violence**		
One	27	75
Two	< 5	< 14
Not reported	5	14
**Relationship of perpetrator to deceased**^**1**^		
Friend or acquaintance	8	23
Current or former intimate partner	8	23
Stranger/unknown to deceased	5	14
Family member	< 5	< 14
Not reported	13	37
**Location of violent incident**		
Road/street/parking area	14	39
Other person’s private residence	12	33
Deceased’s usual place of residence	8	22
Other (water/commercial area)	< 5	< 14
**Day of violent incident**		
Friday	9	25
Sunday	7	19
Saturday	5	14
Monday-Thursday	10	28
Not reported	5	14
**TOXICOLOGY (*n*** **= 23)**		
**Violence-related deaths with available toxicology reports**	23	100
**At least one substance detected**	22	96
Cannabis	16	70
Alcohol	15	65
Other substances^2^	10	43
**Multiple substances detected**	16	70
Alcohol and cannabis	9	39
Multiple substances (excluding alcohol)	6	26

1. Percentages calculated off total number of perpetrators (*n* = 35). For five decedents no information on the perpetrator of violence were reported. 2. Other substances includes: Benzodiazepines, Morphine, Meth/Amphetamines, Antidepressants, Ketamine, Atropine, Ibuprofen, Lignocaine, Metoclopramide, Paracetamol, Phenytoin, Propofol, Citalopram

Information on the person(s) who perpetrated the violence was limited and inconsistently reported. There were 35 perpetrators of violence across the 31 violence-related deaths where some information on the perpetrator(s) was reported. There was most frequently one perpetrator of violence for each death (*n* = 27; 75%), who was usually male (*n* = 32; 91%). For women the perpetrator was often a current or former intimate partner (*n* = 5/8; 63%), and for men, they were commonly a friend or acquaintance (*n* = 6/27; 22%), followed by a stranger (*n* = 5/27; 19%).

### Toxicology of violence-related deaths

Toxicology reports were available for 23 (64%) violence-related deaths. At least one substance was present in the majority of these deaths (*n* = 22; 96%; Table 2), with a median of two substances present (IQR: 1-3) in the toxicology reports. In six (26%) toxicology reports only one substance was identified, which was most frequently alcohol (*n* = 5; 83%), whereas multiple substances were detected in 16 (70%) toxicology reports. Cannabis was the most common substance present across all reports (*n* = 16; 70%), followed by alcohol (*n* = 15; 65%). Other substances detected included benzodiazepines, morphine, and meth/amphetamines (all *n* < 5). The most common co-occurring substances were alcohol and cannabis (*n* = 9; 39%). The toxicology results were similar across sex and Indigenous status.

## Discussion

This is the first study to examine the circumstances and toxicology of violence-related deaths among justice-involved young people. In this large, whole-population cohort from Queensland, Australia, the proportion of deaths from violence (4%) was considerably lower than previous US studies (48-68%) [1, 2], but is comparable to a smaller Australian study that examined deaths after release from youth detention in Victoria (3%) [3]. From 2000 to 2016, the violence-related death rate among those aged 10-44 years (at the end of the study period the oldest person in the cohort was 42 years in age) in the general population in Australia was 1.5 per 100,000 population [21]. Although the proportion of deaths from violence in this cohort was comparatively small, the rate of violence-related death among justice-involved young people (6 deaths per 100,000 person-years) is substantially higher than the rate of death in the Australian general population.

There were clear sex differences in the circumstances of violence-related deaths. Among women, fatal violence was often prefaced by intimate partner violence or relationship breakdown. In comparison, among men, violence-related deaths commonly occurred after an altercation with a friend or acquaintance. Previous studies examining violence-related deaths in this population did not report on the person(s) who perpetrated the violence [1, 3, 4, 22]. Although, our finding is consistent with the gender differences in violence-related deaths in the general population both in Australia [23] and internationally [7]. While there are very few rigorously evaluated violence prevention interventions for young women involved in the youth justice system, a randomised controlled trial involving skill-building exercises and HIV prevention information was found to potentially reduce violence victimisation by helping adult women released from prison make healthier decisions about their relationships [24]. Additional evidence-based interventions that meet the needs of justice-involved young women and support them to stay safe from violence appear indicated and should be paired with universal policies that aim to foster healthy norms and attitudes towards women.

Most of the deaths from violence in the cohort involved the use of a weapon. The use of any weapon in a violent event is associated with an increase in severity of injuries [25]. Reducing the use of weapons may reduce the severity of injuries and risk of death sustained from violent events. Consistent with patterns of weapon use at the population level in Australia [26] and the United Kingdom (UK) [27], the most commonly reported weapon used in the cohort was a knife. There is limited evidence on effective interventions to reduce knife violence. A systematic review of knife crime among young people in the UK found that adverse childhood experiences, poor mental health, previous violence victimisation, having peers that carry knives, economic deprivation and unemployment were connected to young people carrying knives and perpetrating knife violence [28]. These factors are also common among young people who have contact with the youth justice system [29]. In our cohort, people who died from violence were commonly not employed, which is also a risk factor for violence victimisation in the general population, particularly for men [30].

Our finding is in contrast to previous US studies, which found that the vast majority of violence-related deaths among young people released from youth detention involved a firearm [1, 22]. This likely reflects the differences in firearm policies and availability between the US and Australia [31]. Limiting the availability and use of firearms through implementing restrictive firearm policies, such as in Australia [32], is associated with a decrease in violence-related deaths at the population level [32]. This difference in firearm policy may also be contributing to the greater number of violence-related deaths in the US cohorts compared to our study cohort.

Indigenous people were disproportionately impacted by violence-related death. This finding has parallels in the US context, where people who identified as Black or Hispanic were more likely to die from violence than other demographic groups [1, 22]. Indigenous people in Australia [33], and Black and Hispanic people in the US [34, 35], are overrepresented in the youth and adult criminal justice systems and in violence-related deaths at the population level. However, these groups are not homogenous, and each has different histories of racial oppression (e.g., colonisation, slavery). Diverting people away from the criminal justice system and redirecting funds towards community-based health and social services may be an opportunity for violence prevention. In Australia, community justice models that include justice-reinvestment programs may both reduce the overrepresentation of Indigenous people in the criminal justice system and reduce violence in Indigenous communities [36].

Although limited by the number of toxicology reports available, cannabis and alcohol were the most frequent substances detected in these violence-related deaths. Additionally, many of the contributing causes of death in this study were substance-related. Although previous studies on this population did not examine the toxicology of violence-related deaths [1, 3, 4, 22], alcohol use disorder during adolescence was identified as a risk factor for violence-related death [1]. Our findings are consistent with the association between alcohol and cannabis use and an increased risk of violence victimisation in the general population in Australia [12] and internationally [8]. Given the high prevalence of substance use disorders among justice-involved young people [10], the provision of targeted and age-appropriate therapeutic interventions to reduce harmful substance use – both in detention and in the community – may improve health and reduce violence victimisation in this population.

### Strengths and limitations

This study used rigorous data linkage methods, a large whole-population cohort, and had an extended follow-up period (up to 23 years). Linking youth justice and coroner’s records improved our ascertainment of violence-related deaths among justice-involved young people, as many of the coroner’s reports did not explicitly mention the deceased’s involvement in the youth justice system [37]. Despite this, the study was limited by the small number of violence-related deaths in the cohort over the study period. While data from coroners’ investigations provided detailed information on violence-related deaths in the cohort, the information available differed between deaths, depending on a third party’s subjective assessment of its relevance, or lack thereof, to the death. Given the preventable nature of these deaths, there was a surprising lack of information reported for some variables (Table 2). Future studies may benefit from linking with police or court records to improve our understanding of the people who perpetrated the violence. Additionally, not all full-text reports were available for each death on the NCIS, which has been noted previously as a limitation of the NCIS [38]. While we linked to national death records, we examined youth justice contact in only one state in Australia (Queensland); it is unknown how representative these youth justice data are of other Australian states.

## Conclusions

This study represents an important first step towards developing violence prevention interventions that are specifically designed for this population. Although, as in the general population, universal approaches that address the structural drivers of violence, such as attitudes towards women, availabilities of weapons, social inequities and racism, are also likely important to preventing these deaths. Given that the vast majority of violence-related deaths among women were in the context of intimate partner violence, interventions that prevent intimate partner violence among justice-involved young women are urgently needed. Therapeutic alcohol and other drug programs may be an effective violence prevention strategy in this population. To be effective, such programs must be available in the community as well as in detention, and must be coordinated with other essential services for this population, notably mental healthcare. There remains an urgent need to reduce the overrepresentation of Indigenous people and people of colour in the youth justice system, and to increase investment in community-based health and social services.

## Supplementary Information

**Additional file 1.**

**Additional file 2.**

## Data Availability

The datasets generated and/or analysed during the current study are not publicly available due to the nature of this research, the participants of this study did not agree for their data to be shared publicly. Queries can be directed to the corresponding author: Melissa Willoughby, mwilloughby@student.unimelb.edu.au.
