# Supplementary material for: Circumstances and toxicology of violence-related deaths among young people who have had contact with the youth justice system: a data linkage study
Source: BMC Public Health. 2021 Dec 3;21:2207. doi: 10.1186/s12889-021-12244-z (PMC8642952; doi:10.1186/s12889-021-12244-z)
Supplement: Supplementary file 1 — Additional file 1. [file 12889_2021_12244_MOESM1_ESM.docx]

48,670 young people were involved in the youth justice system from 30 June 1993 to 1 July 2014

982 (78%) deaths in the community had a closed record on the NCIS

170 (0.3%) people died before the time frame captured by the NCIS^1^

**36 (4%) deaths from violence-related causes**

946 (96%) deaths from other causes

1,261 (3%) people died within the time frame captured by the NCIS^1^

265 (21%) deaths did not have an NCIS record or the coronial investigation was still ongoing

14 (1%) deaths during incarceration

**Supplementary Figure S1.** Identifying violence-related deaths reported to a coroner among justice-involved young people

1. Records from coroner’s death investigations were available on the National Coronial Information System (NCIS) from 1 July 2000 (1 January 2001 for Queensland) to 31 January 2017. Fifteen deaths, which were excluded from the NCIS, occurred in Queensland between 1 July 2000 and 1 January 2001. Only deaths where the coroner’s investigation was complete at time of data extraction were included (closed records).
