# Supplementary material for: Circumstances and toxicology of violence-related deaths among young people who have had contact with the youth justice system: a data linkage study
Source: BMC Public Health. 2021 Dec 3;21:2207. doi: 10.1186/s12889-021-12244-z (PMC8642952; doi:10.1186/s12889-021-12244-z)
Supplement: Supplementary file 2 — Additional file 2. [file 12889_2021_12244_MOESM2_ESM.docx]

**Supplementary Table S1.** Causes of deaths occurring in the community reported to a coroner among justice-involved young people

| **Causes of death^1^** | **Frequency (n)** | **Proportion (%)** |
| --- | --- | --- |
| Violence-related | 36 | 4 |
| Suicide | 363 | 37 |
| Transport accident | 190 | 19 |
| Accidental drug-related deaths | 160 | 16 |
| Non-external causes | 78 | 8 |
| Other external causes | 69 | 7 |
| Unknown | 86 | 9 |
| **Total** | **982** | **100** |
| 1. Modified from Randall, D., Roxburgh, A., Gibson, A., & Degenhardt, L. (2009). Mortality among people who use illicit drugs: A toolkit for classifying major causes of death. Sydney: National Drug and Alcohol Research Centre, University of NSW. | | |
